# Impact of telemedicine in hospital culture and its consequences on quality of care and safety

**DOI:** 10.1590/S1679-45082015GS2893

**Published:** 2015

**Authors:** Milton Steinman, Renata Albaladejo Morbeck, Philippe Vieira Pires, Carlos Alberto Cordeiro Abreu, Ana Helena Vicente Andrade, Jose Claudio Cyrineu Terra, José Carlos Teixeira, Alberto Hideki Kanamura

**Affiliations:** 1 Hospital Israelita Albert Einstein, São Paulo, SP, Brazil.; 2 Hospital Municipal Dr. Moysés Deutsch, São Paulo, SP, Brazil.; 3 Sociedade Beneficente Israelita Brasileira Albert Einstein, São Paulo, SP, Brazil.

**Keywords:** Telemedicine, Safety, Patient safety, Quality of health care, Emergencies

## Abstract

**Objective:**

To describe the impact of the telemedicine application on the clinical process of care and its different effects on hospital culture and healthcare practice.

**Methods:**

The concept of telemedicine through real time audio-visual coverage was implemented at two different hospitals in São Paulo: a secondary and public hospital, *Hospital Municipal Dr. Moysés Deutsch*, and a tertiary and private hospital, *Hospital Israelita Albert Einstein*.

**Results:**

Data were obtained from 257 teleconsultations records over a 12-month period and were compared to a similar period before telemedicine implementation. For 18 patients (7.1%) telemedicine consultation influenced in diagnosis conclusion, and for 239 patients (92.9%), the consultation contributed to clinical management. After telemedicine implementation, stroke thrombolysis protocol was applied in 11% of ischemic stroke patients. Telemedicine approach reduced the need to transfer the patient to another hospital in 25.9% regarding neurological evaluation. Sepsis protocol were adopted and lead to a 30.4% reduction mortality regarding severe sepsis.

**Conclusion:**

The application is associated with differences in the use of health services: emergency transfers, mortality, implementation of protocols and patient management decisions, especially regarding thrombolysis. These results highlight the role of telemedicine as a vector for transformation of hospital culture impacting on the safety and quality of care.

## INTRODUCTION

Survival rates in emergency care vary significantly among hospitals, reflecting in quality of care. Many hospitals do not have the appropriate facilities and necessary skills to provide effective specialist treatment, 24 hours a day, 7 days a week.^(1,2)^

In this scenario, options to solve health problems of critically ill patients are sought, using effective resources to provide quality of care. In this context, telemedicine is as a viable alternative to offer efficient resolution of problems. The World Health Organization (WHO) defines telemedicine as “the offering services related to health care, where the distance is a critical factor”.^(3)^ Telemedicine has the potential to improve quality of care by allowing clinicians in one “control center” to monitor and even care for and perform procedures on patients in multiple locations. Telemedicine experience in other medical specialties fully applies in the field of emergency medicine.^(4,5)^

The Brazilian Ministry of Health decided to create the *Projeto de Apoio ao Desenvolvimento Institucional ao Sistema Único de Saúde* (PROADI-SUS) to improve health services throughout the country. The PROADI-SUS allows some hospitals, classified as “excellent” in terms of quality of care, to fund projects by means of philanthropy.^(6)^ Hence, the *Hospital Israelita Albert Einstein* (HIAE) developed a telemedicine program to enable teams from emergency department and intensive care unit (ICU) of public hospitals, including the *Hospital Municipal Dr. Moysés Deutsch* (HMMD), to receive real-time support from specialist staff in the emergency department and ICU at HIAE.

## OBJECTIVE

To describe the impact of the telemedicine application on the clinical process of care and its different effects on knowledge sharing, productivity, knowledge-oriented culture and quality service in emergency department and intensive care unit of a secondary hospital, located in distant and deprived area of a great urban center in distance area.

## METHODS

In January 2012, the concept of telemedicine (synchronous connection) was implemented between HIAE and HMMD. A Telemedicine Central Command (TCC) located at HIAE was implemented with Endpoint Cisco^™^ VX Clinical Assistance - 97 MXP Cisco^™^ Solution and Medigraf GoWireless^™^ via a dedicated broadband connection of 2Mb.

In the remote hospital HMMD, a mobile Intern MXP ISDN/IP Cisco^™^ and Medigraf GoWireless^™^ were introduced. The wireless network ensures quality signal transmission throughout the adult emergency department and ICU. Radiologic and computed tomography scans were evaluated using Picture Archiving and Communication System (PACS). TCC offers 24 hours a day, 7 days a week coverage provided by the emergency department and ICU consultants and a full range of services and specialties in house, including all medical specialties. A total of 16 physicians were selected to dedicate to the telemedicine program, based on the following characteristics: motivation to participate in the project; specialization in ICU or emergency department; ability to deal with difficulties and propose solutions; and mastery of all service protocols offering support to patients.

Inclusion criteria to access telemedicine were based upon remote ICU or emergency department physician judgment. Every recruited patient, whether transferred or not, was assessed by the central command through teleconference with an experienced consultant. Furthermore, 36 clinical protocols were developed to cover a broad range of conditions commonly observed in ICU and emergency department patients. Among the protocols available, four were considered as the most important due to high prevalence of the disease in the community, and to severity of cases. The protocols included sepsis, acute coronary syndromes, stroke and trauma. They were printed and provided at the emergency department and ICU of HMMD.

Routine data analyzed included patient demographics, source of referral, details of chief complaint and diagnosis, exams and tests performed, throughput times, treatments given and discharge categories. The data specifically collected for each case during teleconsultations included: patient ID, names and grades of staff requesting and providing consultations, chief complaint, date and time of consultation, type of consultation and images discussed, reason for consultation and query to be resolved, time taken and resources used, nature and impact of any technical problems, diagnosis, and referral or treatment advice given.

The use of telemedicine has been included in the decision support algorithms in major emergencies: trauma (especially head trauma), sepsis, stroke, and ST segment elevation myocardial infarction. The project was approved by the Ethics Committee Research of *Hospital Israelita Albert Einstein* under protocol number 708.447, CAAE: 29221014.0.0000.0071.

## RESULTS

From May 2012 to May 2013, a total of 307 patient records from 257 patients were analyzed. The mean age was 57.2 years, 58.6% were male, mean Acute Physiology and Chronic Health Disease Classification System II (APACHE II) score was 22.3, and 161 (52.4%) teleconsultations took place in the emergency department, and 146 (47.6%) in the ICU. The main diagnoses are described in table 1. In 172 (56.1%) of the consultations, a specialist other than TCC staff was needed to attend specific requests.

**Table 1:** Main patients’ diagnosis

Diagnosis	n	%
Stroke	99	38.5
Sepsis	84	32.7
Acute myocardial infarction	22	8.6
Trauma	22	8.6
Cardiac arrest	9	3.5
Acute hepatic failure	6	2.3
Diabetic ketoacidosis	3	1.2
Heart failure	3	1.2
Brain tumor	2	0.8
Pulmonary thromboembolism	2	0.8
Cardiac arrhythmia	2	0.8
Acute mesenteric ischemia	1	0.4
Hemorrhagic shock	1	0.4
Exogenous intoxication	1	0.4

Total	257	100

For 18 patients (7.1%) the telemedicine consultation influenced in making a definite diagnosis, and for 239 patients (92.9%), the consultation contributed to clinical management.

Most teleconferences (70%) occurred in the middle of the day, around 1:30 p.m. The mean hospital length of stay was 13.6 days; 171 patients (66.5%) were discharged from the hospital, intra-hospital mortality was 28.4% (73 patients) and 13 patients (5.1%) were transferred to tertiary hospitals; 10 patients (3.9%) were submitted to major surgical procedures (neurosurgery, heart surgery and liver transplantation). In such cases, we followed Public Health rules (Regulation Health System). Out of all 257 patients, 66 (25.6%) had an invasive procedure indicated after the telemedicine session.

After telemedicine consultation, changing antibiotic therapy was suggested in 39 patients (15.1%). Among the 11 patients submitted to thrombolysis due to stroke, the mean time from onset of symptoms to beginning of the procedure was 163.2 minutes; the mean National Institutes of Health (NIH) stroke scale was 14.7 before the thrombolysis and 12.7 at the end of the procedure. There was one case (9.1%) of symptomatic intracranial hemorrhage after the procedure. After the implementation of the telemedicine program, all patients admitted to the emergency department, with neurological diagnosis, eligible to be transferred to a tertiary center for a neurological or neurosurgical evaluation, were submitted to a telemedicine consult with a neurologist of the HIAE staff. With this procedure, the external neurological evaluations decreased by 25.9% after one year of telemedicine program implementation (355 before telemedicine *versus* 263 after telemedicine).

Comparing 1 year before and 1 year after telemedicine implementation regarding acute myocardial infarction, severe sepsis, ischemic and hemorrhagic stroke we did not find a significant difference in terms of hospital mortality (Table 2). On the other hand, we could observe a trend toward a drop in hospital mortality, when comparing patients submitted to the telemedicine consultations to those not using this technology, after the program implementation (Table 3).

**Table 2:** Hospital mortality 1 year before and 1 year after implementation of the telemedicine program

Diagnosis	Mortality	
	1 year before telemedicine program	1 year after telemedicine program
	n (%)	n (%)
AMI	240 (17)	207 (14)
Septic shock	362 (65.7)	417 (67.9)
Ischemic stroke	48 (50)	80 (43.8)
Hemorrhagic stroke	47 (23.4)	78 (27.8)

AMI: acute myocardial infarction.

**Table 3:** Hospital mortality one year after the implementation of the telemedicine program, comparing cases of patients submitted or not to telemedicine consultation

Diagnosis	Mortality	
	Without telemedicine program	With telemedicine program
	n (%)	n (%)
AMI	194 (14.4)	13 (7.6)
Septic shock	375 (70.9)	42 (40.4)
Ischemic stroke	27 (75.6)	53 (32.1)
Hemorrhagic Stroke	46 (36.9)	32 (15.6)

AMI: acute myocardial infarction.

Over a 12-month period, there were 36 TM sessions regarding ischemic stroke. When connecting by TM, the local physicians evaluated the patient’s condition and found thrombolytic therapy to be a possible option in 11.2%.

## DISCUSSION

The ultimate purpose of any medical care is to maintain or improve health and well-being. Thus, how clinical applications of telemedicine interfere in quality of care and its outcomes is a central question to evaluate any health service. Quality of care is “the degree to which health care services for individuals and populations increase the likelihood of desired health outcomes and are consistent with current professional knowledge”.^(7)^

Rigorous evaluation processes play a vital role in the progress of any medical field, and telemedicine is not different. Conducting evaluations and disseminating results may be particularly important to the field of telemedicine given the scarcity of empirical evidence on its use. These evaluations can help generate reliable data to develop national telemedicine policy and strategy, streamline telemedicine implementation, and inform the potential for enhancement and transferability of telemedicine projects.

One prevalent barrier reported all over the world when implementing a telemedicine program was an organization culture not used to share knowledge and skills among professionals and patients in remote locations, via telemedicine. This challenge to change management does not dependent on the country income, available resources, or regional need for telemedicine solutions. Considering the adoption of telemedicine systems requires the acceptance of users involved in the process, this finding may indicate a lack of awareness or discomfort in using of telemedicine systems.^(8)^

Physicians are important gatekeepers in telemedicine adoption and diffusion, and local physician endorsement is a relevant requirement for the success of telemedicine programs, especially regarding emergency department and ICU. The methods that promote interaction among physicians, build trust among providers, and increase awareness of successful treatments via telemedicine and of mutually agreed indications for teleconsultations, help changing attitudes and enhance its utilization.^(5,8)^

Telemedicine is emerging as an increasingly sought-after tool for addressing some of the challenges and changes within the emergency and ICU care, including poor physician access in remote areas and high demand for specialists in both rural and urban areas.

The clinical effects of telemedicine applications can be measured and compared at several levels. One may, *e.g., *look for effects on the process of care or for effects on the outcomes of care or both.^(9)^ In a discussion of the impact of diagnostic technologies, Fineberg et al.^(10)^indicated several process and outcome dimensions, which might appropriately be assessed by evaluators. These dimensions include: technical capacity (whether a technology is safe and accurate) and diagnostic accuracy (if a technology contributes to a correct diagnosis); diagnostic impact (whether a technology provides useful diagnostic data to make diagnosis; for instance, if a presential visit is still necessary after the telemedicine consultation); therapeutic impact (whether a technology influences patient management or therapy), and patient outcome (whether a technology improves patients’ health conditions and well-being) The first four dimensions involve care processes.

It is very important to mention that the best telemedicine impact, as to diagnosis or therapy, relates to the interval between hospital admission and teleconsultation. In our experience, we identified long time intervals despite severity of the cases.

The experience led us to develop evidence-based protocols but tailored to local constraints such as use of thrombolytics in cases of acute myocardial infarction when angioplasty is not available. We have created up to 36 protocols regarding most common ICU and emergency department issues.

By including telemedicine in algorithm decision support, it was possible to verify some cultural changes in organization and its consequences on quality and safety of care.

We identified two main contribution related to telemedicine: diagnosis or clinical management. There were many different contributions depending upon the reason of the telemedicine access and related disease. In fact we identified positive aspects in most cases. Concerning sepsis protocol, *e.g.,* changing the antibiotic therapy was the most common suggestion. It is important to emphasize the importance of proactive behavior of the physician responsible to answer the consultation. At minimum, he might check and re-check diagnosis, therapeutic plans and even could also identify opportunities to reinforce deep venous thrombosis prevention in high-risk patients. Telehealth applications have been conceived, developed, and deployed in a variety of clinical settings; yet the body of evidence supporting their use has been slow to evolve. It is important to mention that once a relation is established between two sites, the impact is related to the frequency of the access. We may suppose that at some point, knowledge transfer was done and the quality of health may reach a similar level in both hospitals and this will be the definite success of the program.

There are reports in the literature confirming that physicians described being more confident in treating acute and complex diseases and higher satisfaction with their jobs. Physicians reported that the telehealth sessions helped them to network socially with other physicians. Medical practices also had lower turnover rates among nurses and other clinic staff. The investigators learned that the practice staff enjoyed interacting with peers at other practices and felt connected to their profession in a way they had not prior to the implementation of the telehealth network.^(9)^

The most effective treatment for acute ischemic stroke is rapid reperfusion. Current recommendations and drug labeling limit the use of intravenous tissue plasminogen activator (tPA) in the United States to within 3 hours of the time the patient was last seen well (or had witnessed onset of symptoms). The main barrier to increasing treatment among those patients arriving within 3 hours is physician reluctance to deliver the therapy in the absence of available acute stroke expertise around the clock.^(11)^

Another reason for reluctance to use intravenous tPA in acute stroke was related to physician fears of side effects and liability. In one survey, 40% of emergency physicians indicated they would not use intravenous tPA, and most mentioned the risk of intracerebral hemorrhage as the reason.^(12)^ Reluctance to administer tPA at hospitals that have not made an institutional commitment to acute stroke care, including the rapid provision of neurological and radiological expertise on demand, is reasonable, because several reports suggested that complication rates may be higher in inexperienced facilities. Reassuringly, some studies showed that training and implementation of stroke teams, made the complication rates return to expected and acceptable levels.^(11,12)^

Before the implementation of telemedicine, thrombolysis had never been used in cases of stroke in HMMD. The professionals might create a sense and a shared repertoire of knowledge when treatment is discussed and carried out. Hereby, more frequent use of telemedicine consultations can lead to knowledge exchanges and a greater number of thrombolytic treatments made by confident professionals.

Although important differences comparing pre- and post-telemedicine implementation were observed, we did not apply any statistical analysis. Comparisons of clinical interventions or programs should be adjusted statistically to account for differences in patient risk factors. Further studies are imperative to confirm this hyphotesis.

One of the characteristics of modern medicine is the propensity to refer the patients to a specialized service. Conversely, professional and economical reasons do not allow every medical center to offer all possible services. Regarding emergency medicine and trauma, the process of sending the patient to another hospital might be problematic. In most Western countries, the trauma physicians are general surgeons who frequently have to deal with all injuries in hospitals without neurosurgical service. Esposito et al.,^(13)^in a retrospective study on a broad cohort (based on the National Trauma Database - NTDB) showed that the actual need for emergency intervention by a neurosurgeon is very rare. In fact, another reason for transfer to a tertiary center, despite the clinical picture, relates to physician’s fear of liability on treating patients without neurosurgical coverage. Alert head trauma patients can be selectively hospitalized in a hospital without neurosurgical service. The selection of the patients can be based on clinical-radiologic algorithm or on teleconsultation. The latter approach allows for better and more effective selection, thus reducing the need to transfer the patient to another hospital.^(14)^

Despite evidence to support efficacy of early goal-directed therapy for resuscitation of patients with severe sepsis and septic shock in the emergency department, implementation remains incomplete. Knowledge gaps and procedural hurdles identified by the telemedicine sessions may play a role to inform both educational and process components of an initiative to improve sepsis care in the emergency department and ICU.

It is interesting to note that in many situations, even after the inclusion of telemedicine in decision algorithms, the tool was not used. There is a natural resistance by medical staff. One objective (stated or not-stated) should be to maximize utilization. If people do not use the program, support will erode. If many people use the system frequently, support will be much easier to achieve. A number of our findings merit further discussion. Our results suggest that staff acceptance of tele-ICU coverage is generally high although team members are concerned about the implementation and routine use of this application. It is very important to involve staff prior to implementation and mitigate conflicts, like liability concerns, extra workload and failure to recognize when this tool is needed. A consistent characteristic of unsuccessful telemedicine programs is that they saw themselves as somehow separate from the overall organization and had independent objectives. These programs lost support over time or were relegated to a minor and often experimental role in care delivery.^(15,16)^

Before the implementation of the resource, many care practices were linked to medical decision making, even after attempts to improve processes, whether in the underutilization of, by not adopting protocols or transfer as a gauge. Thus, when comparing the results between patients who used this feature or not, there is a clear trend towards better prognosis and reduced transfers. This is due to improved processes and greater involvement and commitment of the multidisciplinary staff.

Telemedicine tool was not directly applied to the patient. This study essentially involved physician-physician relationship, based on a clinical problem relate to the patient. This study did not included questionnaire regarding patient satisfaction. We will conduct future studies for this purpose.

Telemedicine is a tool not a goal and it needs to solve a real problem. Medicine is still about people, patients, quality of service and processes. Ensuring continuity of care in a telemedicine environment presents unique challenges. Research is needed to provide evidence of cost efficiencies and improved quality of care through the use of telemedicine for specific diseases. The study presented initial encouraging results, the program proved to be useful in helping diagnosis, conducting critical cases and transferring specialized medical knowledge to hospitals with shortage of human and technical resources.

The full potential of telemedicine will only be realized through change in medical culture and attitudes. In a system of this nature, knowledge sharing took place and established a new paradigm in healthcare delivery. Healthcare organizations can render high quality services from developed areas to less developed locations.

### Limitation

Our study has some important limitations. We made a project with vision of telemedicine as a platform for more comprehensive telemedicine systems treating many conditions in addition to stroke in areas in which access to specialty care has been an important barrier to care delivery. This is a descriptive study, analyzing the initial impact of the implementation of the telemedicine program in the quality and safety of clinical practice in a community hospital in São Paulo (SP), Brazil. Proper interpretation of patient outcomes data requires good information on patient characteristics, in particular, their health status. The comparisons of clinical interventions or programs should be adjusted statistically to account for differences in patient risk factors. Although a trend toward hospital mortality reduction rate was observed in the group of patients submitted to the telemedicine consultation, patients were not compared regarding severity indexes. It might be difficult to conduct a prospective randomized study comparing two groups of patients, concerning ethical and logistical issues. One alternative could include a comparison between two similar hospitals, one containing telemedicine, stratifying the same group of patients and diseases.

## CONCLUSION

This paper aimed to introduce a conceptual framework and gathered some data to prove and show how the implementation of telemedicine can play significant roles in the quality and quantity of services, especially related to knowledge that can be can transferred from all levels of health network. The application is associated with differences in the use of health services: emergency transfers, mortality, implementation of protocols and patient management decisions, especially regarding thrombolysis. These results highlight the role of telemedicine as a vector for transforming hospital culture, with impact on safety and quality of care.
