# Supplementary material for: Impact of telemedicine in hospital culture and its consequences on quality of care and safety
Source: Einstein (Sao Paulo). 2015 Oct-Dec;13(4):580–6. doi: 10.1590/S1679-45082015GS2893 (PMC4878634; doi:10.1590/S1679-45082015GS2893)
Supplement: Translation

# Translation: Impacto da telemedicina na cultura hospitalar e suas consequências na qualidade e segurança do cuidado

**Authors:** Milton Steinman, Renata Albaladejo Morbeck, Philippe Vieira Pires, Carlos Alberto Cordeiro Abreu, Ana Helena Vicente Andrade, Jose Claudio Cyrineu Terra, José Carlos Teixeira, Alberto Hideki Kanamura

**Affiliations:** 1 Hospital Israelita Albert Einstein, São Paulo, SP, Brasil.; 2 Hospital Municipal Dr. Moysés Deutsch, São Paulo, SP, Brasil.; 3 Sociedade Beneficente Israelita Brasileira Albert Einstein, São Paulo, SP, Brasil.

**Keywords:** Telemedicina, Segurança, Segurança do paciente, Qualidade da assistência a saúde, Emergência

## Abstract

**Objetivo:**

Analisar o impacto da aplicação de telemedicina no processo clínico de cuidado e seus diferentes efeitos sobre a cultura hospitalar e na prática assistencial.

**Métodos:**

O conceito de telemedicina, por meio de uma cobertura integral em tempo real, foi implementada em dois hospitais diferentes em São Paulo, um secundário e público, o Hospital Municipal Dr. Moysés Deutsch, e um terciário e privado, o Hospital Israelita Albert Einstein.

**Resultados:**

Os dados foram obtidos a partir de 257 teleconsultas registradas ao longo de um período de 12 meses e comparados a igual período antes da implementação. Em 18 pacientes (7,1%), a telemedicina influenciou no diagnóstico e, para 239 pacientes (92,9%), a consulta contribuiu para o manejo clínico. Após a implementação da telemedicina, o protocolo de trombólise para acidente vascular cerebral foi aplicado em 11% dos pacientes com acidente vascular cerebral. A introdução da telemedicina reduziu a necessidade de transferir pacientes para avaliação neurológica externa em 25,9%. O protocolo de sepse foi adotado, sendo observada redução da mortalidade em 30,4% nos casos de sepse grave.

**Conclusão:**

A aplicação da telemedicina está associada com diferenças na utilização dos serviços de saúde: transferências, mortalidade, implementação de protocolos e apoio à decisão médica, especialmente em relação à trombólise. Estes resultados destacam o papel da telemedicina como um vetor de transformação da cultura hospitalar e seu impacto sobre a segurança e na qualidade assistencial.

## INTRODUÇÃO

As taxas de sobrevida no cuidado de emergência variam significativamente entre os hospitais, refletindo na qualidade do cuidado. Muitos hospitais não têm as instalações adequadas e nem as habilidades necessárias para oferecer um tratamento especializado efetivo, durante 24 horas por dia, 7 dias por semana.^(1,2)^

Nesse cenário, buscam-se opções para resolver os problemas de saúde de pacientes graves, por meio de recursos efetivos para prestar um cuidado com qualidade. Nesse contexto, a telemedicina é uma alternativa viável para oferecer uma resolução eficiente desses problemas. A Organização Mundial da Saúde (OMS) define a telemedicina como “a oferta de serviços relacionados à saúde, onde a distância é um fator crucial”.^(3)^ A telemedicina tem o potencial para melhorar a qualidade do cuidado, ao permitir que os médicos em um “centro de controle” monitorem, atendam e realizem alguns procedimentos em pacientes que estão em diferentes lugares. A experiência da telemedicina em outras especialidades médicas aplica-se integralmente ao campo da medicina de emergência.^(4,5)^

No Brasil, o Ministério da Saúde decidiu criar o Projeto de Apoio ao Desenvolvimento Institucional ao Sistema Único de Saúde (PROADI-SUS) para melhorar os serviços de saúde em todo o país. O PROADI-SUS permite que alguns hospitais classificados como “excelentes” em termos da qualidade da assistência financiem projetos por meio da filantropia.^(6)^ Assim, o Hospital Israelita Albert Einstein (HIAE) desenvolveu um programa que permite que as equipes do pronto-socorro e da unidade de terapia intensiva (UTI) de hospitais públicos, incluindo o Hospital Municipal Dr. Moysés Deutsch (HMMD), recebam apoio em tempo real de seus especialistas.

## OBJETIVO

Descrever o impacto da aplicação da telemedicina no processo clínico de cuidado e seus diferentes efeitos sobre o compartilhamento de conhecimento, a produtividade, a cultura voltada para o conhecimento, e um serviço de qualidade no departamento de emergência e na unidade de terapia intensiva de um hospital secundário, localizado em uma área distante e carente em um grande centro urbano na área de distância.

## MÉTODOS

Em janeiro de 2012, o conceito de telemedicina (conexão sincrônica) foi implementado entre o HIAE e o HMMD. Foi implementado um Comando Central de Telemedicina (CCT) localizado no HIAE, utilizando-se a solução *Endpoint Cisco* ^®^ *VX Clinical Assistance - 97 MXP Cisco* ^®^ *Solution e o Medigraf GoWireless* ^®^, por meio de uma conexão de banda larga dedicada de 2Mb.

No hospital remoto, o HMMD, foi introduzido um Intern MXP ISDN/IP Cisco^® ^móvel e um Medigraf GoWireless^®^. A rede sem fio garante uma transmissão de sinal de qualidade pelo pronto-socorro e pela UTI adultos. Exames radiológicos e de tomografia computadorizada foram avaliados utilizando um Sistema de Comunicação e Arquivamento de Imagens (SCAI). O CCT oferece cobertura 24 horas por dia, 7 dias por semana, pelos consultores no pronto-socorro e na UTI, além de uma linha completa de serviços e especialidades internas, incluindo todas as especialidades médicas. Dezesseis médicos foram selecionados para se dedicarem ao programa de telemedicina, tendo as seguintes características: motivação para participação no projeto; especialização em medicina intensiva ou de emergência; capacidade para lidar com dificuldades e propor soluções; e domínio de todos os protocolos do serviço, oferecendo suporte aos pacientes.

Os critérios de inclusão para acesso à telemedicina basearam-se no julgamento do médico de UTI ou pronto-socorro remoto. Cada paciente selecionado, tendo ele sido transferido ou não, foi avaliado pelo comando central por uma teleconferência com um consultor experiente. Além disso, 36 protocolos clínicos foram desenvolvidos, os quais cobriam uma ampla gama de doenças encontradas comumente na UTI e no pronto-socorro. Dentre os protocolos disponíveis, quatro foram considerados os mais importantes, devido à alta prevalência da doença na comunidade e à gravidade dos casos. Os protocolos incluíam sepse, síndromes coronarianas agudas, acidente vascular cerebral, hipotermia terapêutica após parada cardíaca e trauma. Tais protocolos foram impressos e oferecidos no pronto-socorro e na UTI do HMMD.

Os dados de rotina analisados incluíam dados demográficos dos pacientes, origem do encaminhamento, detalhes da queixa principal e diagnóstico, exames realizados, velocidades de processamento, tratamentos administrados e categorias de alta. Os dados especificamente coletados para cada caso durante as teleconsultas incluíram RG do paciente, nomes e categorias dos profissionais que solicitaram e realizaram as consultas, queixa principal, data e horário de consulta, tipo de consulta e exames de imagem discutidos, motivo da consulta e dúvida a ser solucionada, tempo gasto e recursos utilizados, natureza e impacto de qualquer problema técnico, diagnóstico e encaminhamento ou orientação e tratamento.

O uso da telemedicina foi incluído nos algoritmos de suporte à decisão nas principais emergências: trauma (especialmente trauma craniano), sepse, acidente vascular cerebral e infarto do miocárdio com elevação do segmento ST. O projeto foi aprovado pelo Comitê de Ética em Pesquisa do Hospital Israelita Albert Einstein número do protocolo: 708.447, CAAE: 29221014.0.0000.0071.

## RESULTADOS

De maio de 2012 a maio de 2013, foi analisado um total de 307 prontuários de 257 pacientes. A média de idade foi de 57,2 anos de idade, 58,6% eram do sexo masculino. O escore *Acute Physiology and Chronic Health Disease Classification System II* (APACHE II) médio foi de 22,3, 161 (52,4%) teleconsultas foram realizadas no pronto-socorro e 146 (47,6%) foram realizadas na UTI. Os principais diagnósticos estão descritos na tabela 1. Em 172 (56,1%) consultas, foi necessário um especialista de fora do CCT para atender a demandas específicas.

**Tabela 1:** Principal diagnóstico dos pacientes

Diagnóstico	n	%
Acidente vascular cerebral	99	38,5
Sepse	84	32,7
Infarto agudo do miocárdio	22	8,6
Trauma	22	8,6
Parada cardíaca	9	3,5
Falência hepática aguda	6	2,3
Cetoacidose diabética	3	1,2
Insuficiência cardíaca	3	1,2
Tumor cerebral	2	0,8
Embolia pulmonar	2	0,8
Arritmia cardíaca	2	0,8
Isquemia mesentérica aguda	1	0,4
Choque hemorrágico	1	0,4
Intoxicação exógena	1	0,4

Total	257	100

Para 18 pacientes (7,1%), a consulta por telemedicina influenciou na conclusão do diagnóstico, e para 239 pacientes (92,9%), a consulta contribuiu para o manejo clínico.

A maioria das teleconferências (70%) ocorreu no meio do dia, aproximadamente às 13h30. A duração média da estadia hospitalar foi de 13,6 dias, 171 pacientes (66,5%) tiveram alta hospitalar, a mortalidade intra-hospitalar foi de 28,4% (73 pacientes) e 13 pacientes (5,1%) foram transferidos para hospitais terciários; 10 pacientes (3,9%) foram submetidos a grandes procedimentos cirúrgicos (neurocirurgia, cirurgia cardíaca e transplante de fígado). Nesses casos, seguimos as regras de Saúde Pública (Sistema Regulatório de Saúde). Dentre todos os 257 pacientes, 66 (25,6%) tiveram a indicação de um procedimento invasivo após a sessão de telemedicina.

Após a consulta por telemedicina, sugeriu-se a alteração da antibioticoterapia para 39 pacientes (15,1%). Dentre os 11 pacientes submetidos à trombólise no acidente vascular cerebral, o tempo médio do início dos sintomas até o início do procedimento foi de 163,2 minutos, a pontuação média na Escala de acidente vascular cerebral do *National Institutes of Health* (NIH) foi de 14,7 antes da trombólise e de 12,7 ao final do procedimento. Houve um caso (9,1%) de hemorragia intracraniana sintomática após o procedimento. Após a implementação do programa de telemedicina, todos os pacientes admitidos no pronto-socorro com diagnóstico neurológico e elegíveis para uma transferência para um centro terciário para que pudessem ser submetidos a uma avaliação neurológica ou neurocirúrgica foram submetidos a uma consulta por telemedicina com um neurologista da equipe do HIAE. Com esse procedimento, as avaliações neurológicas externas caíram 25,9% após 1 ano da implementação do programa de telemedicina (355 antes da telemedicina *versus* 263 após a telemedicina).

Comparando 1 ano antes e 1 ano depois da implementação da telemedicina com relação a infarto agudo do miocárdio, sepse severa e acidente vascular cerebral isquêmico e hemorrágico, não encontramos diferença significativa em termos de mortalidade hospitalar (Tabela 2). Por outro lado, pudemos observar uma tendência em direção à redução da mortalidade hospitalar quando comparamos pacientes submetidos às consultas por telemedicina com aqueles que não fizeram uso dessa tecnologia, após a implementação do programa (Tabela 3).

**Tabela 2:** Mortalidade hospitalar 1 ano antes e 1 ano depois da implementação do programa de telemedicina

Diagnóstico	Mortalidade	
	Um (1) ano antes do programa de telemedicina	Um (1) ano após o programa telemedicina
	n (%)	n (%)
IAM	240 (17)	207 (14)
Choque séptico	362 (65,7)	417 (67,9)
Acidente vascular cerebral isquêmico	48 (50)	80 (43,8)
Acidente vascular cerebral hemorrágico	47 (23,4)	78 (27,8)

IAM: infarto agudo do miocárdio.

**Tabela 3:** Mortalidade hospitalar 1 ano antes e 1 ano após a implementação do programa de telemedicina

Diagnóstico	Mortalidade	
	Antes do programa telemedicina	Após o programa telemedicina
	n (%)	n (%)
IAM	194 (14,4)	13 (7,6)
Choque séptico	375 (70,9)	42 (40,4)
Acidente vascular cerebral isquêmico	27 (75,6)	53 (32,1)
Acidente vascular cerebral hemorrágico	46 (36,9)	32 (15,6)

IAM: infarto agudo do miocárdio.

Durante um período de 12 meses, com relação a acidentes vasculares cerebrais isquêmicos, as sessões de telemedicina foram utilizadas 36 vezes. Por meio da conexão pela telemedicina, os médicos locais avaliaram a doença do paciente e descobriram que o tratamento trombolítico era uma possível em 11,2% dos casos.

## DISCUSSÃO

A principal finalidade de qualquer cuidado médico é manter ou melhorar a saúde e o bem-estar. Portanto, a forma como as aplicações clínicas da telemedicina afetam a qualidade do cuidado e os seus resultados é uma questão avaliatória central para qualquer serviço de saúde. A qualidade do cuidado é “o ponto até o qual os serviços de saúde para indivíduos e populações aumentam a probabilidade dos desfechos de saúde desejados e o ponto até o qual são consistentes com o conhecimento profissional atual”.^(7)^

Processos rigorosos de avaliação têm um papel essencial no progresso de qualquer área médica, e o mesmo ocorre com a telemedicina. A realização de avaliações e a disseminação de resultados podem ser especialmente importantes para o campo da telemedicina, devido à escassez de evidências empíricas de sua utilização. Essas avaliações podem ajudar a gerar dados confiáveis para desenvolver políticas e estratégias nacionais de telemedicina, para otimizar a implementação da telemedicina e para informar o potencial de melhoria e transferência de projetos de telemedicina.

Uma das barreiras prevalentes relatadas globalmente na implementação de um programa de telemedicina foi uma cultura organizacional desacostumada com o compartilhamento e a troca de conhecimentos e habilidades entre profissionais e pacientes em locais remotos por meio da telemedicina. Esse desafio de mudança na gestão não depende da renda do país, dos recursos disponíveis ou da necessidade regional de soluções em telemedicina. Já que a adoção de sistemas de telemedicina exige a aceitação dos usuários envolvidos no processo, esse achado pode indicar uma falta de conscientização ou um desconforto quanto ao uso dos sistemas de telemedicina.^(8)^

Os médicos são representantes importantes na adoção e na difusão da telemedicina, e o aval dos médicos locais é um pré-requisito importante para o sucesso de tais programas, especialmente quando se trata da emergência e da UTI. Métodos que promovem a interação médico-médico, que aumentam a confiança entre os prestadores e que aumentam a conscientização sobre os tratamentos bem-sucedidos por meio telemedicina e sobre as indicações de comum acordo da telemedicina ajudam todos a mudar atitudes e a aumentar sua utilização.^(5,8)^

A telemedicina tem emergido como uma ferramenta cada vez mais buscada para lidar com alguns dos desafios e das mudanças no cuidado em emergência e UTI, incluindo o acesso insatisfatório a médicos em áreas remotas e a alta demanda por especialistas, tanto em áreas rurais quanto urbanas.

Os efeitos clínicos das aplicações da telemedicina podem ser medidos e comparados em vários níveis. Um deles, por exemplo, pode investigar os efeitos sobre o processo de cuidado ou os efeitos dos desfechos do cuidado, ou ambos.^(9)^ Em uma discussão sobre o impacto das tecnologias diagnósticas, Fineberg et al.^(10)^ distinguiram várias dimensões de processo e resultado, que podem ser adequadamente examinadas por avaliadores. Essas dimensões incluem: capacidade técnica (se uma tecnologia é segura e precisa) e precisão diagnóstica confiável (se uma tecnologia contribui para um diagnóstico correto); impacto diagnóstico (se uma tecnologia oferece dados diagnósticos úteis para alcançar um diagnóstico, por exemplo, após a consulta por telemedicina, ainda é necessária uma consulta presencial?); impacto terapêutico (se uma tecnologia influencia no manejo do paciente ou na terapia) e desfecho do paciente (se uma tecnologia melhora a saúde e o bem-estar do paciente). As quatro primeiras dimensões envolvem o processo de cuidado.

É muito importante mencionar que o melhor impacto da telemedicina, com relação ao diagnóstico ou à terapêutica, está relacionado ao intervalo entre a admissão hospitalar e a teleconsulta. De acordo com nossa experiência, identificamos grandes intervalos, apesar da gravidade dos casos.

A experiência nos levou a desenvolver protocolos baseados em evidências, porém, feitos sob medida para as limitações locais, tais como o uso de trombolíticos em casos de infarto agudo do miocárdio quando a angioplastia não estivesse disponível. Criamos até 36 protocolos relacionados aos principais problemas do pronto-socorro e da UTI.

Com a inclusão da telemedicina nos algoritmos de suporte à decisão foi possível verificar algumas mudanças culturais na organização e suas consequências sobre a qualidade e a segurança assistencial.

Identificamos duas principais contribuições relacionadas à telemedicina: diagnóstico ou manejo clínico. Houve várias contribuições dependendo do motivo para o acesso à telemedicina e da doença relacionada. Na verdade, identificamos contribuições na maioria dos casos. Com relação ao protocolo para sepse, por exemplo, o esquema de alteração dos antibióticos foi a sugestão mais comum. É importante enfatizar a importância do comportamento proativo do médico responsável por responder à consulta. No mínimo, o médico poderia verificar, e verificar novamente o diagnóstico e os planos terapêuticos, além de também até identificar oportunidades de reforçar a prevenção da trombose venosa profunda em pacientes de alto risco. Aplicações de telessaúde foram concebidas, desenvolvidas e utilizadas em vários cenários clínicos; entretanto, o conjunto de provas que fundamenta sua utilização desenvolveu-se lentamente. É importante mencionar que, após uma relação ter sido estabelecida entre os dois locais, o impacto está relacionado à frequência do acesso. Podemos supor que, em certo momento, a transferência de conhecimento ocorreu e que a qualidade da saúde pôde alcançar um nível semelhante em ambos os hospitais, e isso representa o sucesso definitivo do programa.

Há relatos na literatura confirmando que os prestadores médicos relataram sentirem-se mais confiantes no tratamento de doenças agudas e complexas, e maior satisfação com seu trabalho. Os médicos relataram que as sessões de telessaúde ajudaram-nos a interagir socialmente com outros médicos. As clínicas também relataram taxas mais baixas de rotatividade entre enfermeiros e outros membros da equipe clínica. Os pesquisadores descobriram que a equipe clínica gostou de interagir com colegas em outras clínicas e sentiu-se ligada à sua profissão de uma forma que não se sentia antes da implementação da rede de telessaúde.^(9)^

O tratamento mais eficiente para o acidente vascular cerebral isquêmico agudo é a reperfusão rápida. Recomendações atuais e a rotulagem de medicamentos limitam o uso do ativador do plasminogênio tecidual intravenoso (tPA) nos Estados Unidos a até 3 horas a partir do horário em que o paciente foi visto sentindo-se bem (ou em que foi observado o início dos sintomas). A principal barreira para o aumento do tratamento entre esses pacientes que chegam dentro das 3 horas é a relutância, por parte dos médicos, em aplicar a terapia na ausência de especialistas em acidente vascular cerebral agudo disponíveis 24 horas por dia.^(11)^

Outro motivo para a relutância em utilizar os tPA intravenosos no acidente vascular cerebral agudo esteve relacionado aos receios dos médicos quanto a efeitos colaterais e responsabilidade. Em um estudo, 40% dos médicos de emergência indicaram que não usariam tPA intravenoso, sendo que a maioria citou o risco de hemorragia intracerebral como o motivo.^(12)^ A relutância em administrar tPA em hospitais que não assumiram o compromisso de prestar cuidados para o acidente vascular cerebral agudo, incluindo o fornecimento rápido de *expertise* em neurologia e radiologia sob demanda, é razoável, já que vários relatórios sugerem que as taxas de complicação podem ser mais altas em locais com pouca experiência. Felizmente, estudos mostraram que, com o treinamento e a implementação de equipes de acidente vascular cerebral, as taxas de complicação retornam aos níveis esperados e aceitáveis.^(11,12)^

Antes da implementação da telemedicina, a trombólise nunca havia sido utilizada em casos de acidente vascular cerebral no HMMD. Por meio do diálogo e da telemedicina, as discussões médicas surgem e os problemas são resolvidos. Os profissionais podem criar um senso e um repertório compartilhado de conhecimento quando o tratamento é discutido e posto em prática. Desse modo, o uso mais frequente de consultas por telemedicina pode levar à troca de conhecimentos e a um maior número de tratamentos trombolíticos administrados por profissionais confiantes.

Apesar de termos verificado diferenças importantes na comparação pré e pós-implementação da telemedicina, não foi aplicada nenhuma análise estatística. Comparações de intervenções ou programas clínicos devem ser ajustadas estatisticamente para levar em conta diferenças nos fatores de risco dos pacientes. Estudos adicionais são necessários para confirmar essa hipótese.

Uma das características da medicina moderna é a propensão a encaminhar os pacientes para um serviço especializado. Por outro lado, razões profissionais e econômicas não permitem que todos os centros médicos ofereçam todos os serviços possíveis. Com relação à medicina de emergência e trauma, o processo de encaminhamento do paciente para outro hospital pode ser problemático. Na maioria dos países ocidentais, os traumatologistas são cirurgiões gerais que frequentemente têm de lidar com todas as lesões em hospitais sem um serviço de neurocirurgia. Em seu estudo retrospectivo com uma ampla coorte (baseado no Banco de Dados Nacional sobre Trauma, NTDB - *National Trauma Database*), Esposito et al.^(13)^ mostraram que a necessidade real de uma intervenção de emergência por parte de um neurocirurgião é muito rara. Na verdade, outro motivo para a transferência para um centro terciário, apesar do quadro clínico, esteve relacionado aos receios dos médicos quanto à responsabilidade pelo tratamento de pacientes sem cobertura neurocirúrgica. Pacientes alertas com trauma craniano podem ser hospitalizados seletivamente sem um serviço de neurocirurgia. A seleção dos pacientes pode ser baseada em um algoritmo clínico-radiológico ou em uma teleconsulta. Essa abordagem permite uma seleção melhor e mais efetiva, reduzindo, portanto, a necessidade de transferir o paciente para outro hospital.^(14)^

Apesar de haver indícios que apoiam a eficácia da terapia precoce orientada para objetivos na ressuscitação de pacientes com sepse severa e em choque séptico no departamento de emergência, a implementação permanece incompleta. Falhas no conhecimento e obstáculos processuais identificados pelas sessões de telemedicina podem ter um papel informativo nos componentes educacional e processual de uma iniciativa para melhorar o cuidado da sepse no departamento de emergência e na UTI.

É interessante notar que, em várias situações, mesmo após a inclusão da telemedicina nos algoritmos de decisão, a ferramenta não foi utilizada. Há uma resistência natural por parte da equipe médica. Um objetivo (explícito ou implícito) deveria ser maximizar a utilização. Se as pessoas não utilizam o programa, o suporte se deteriora. Se muitas pessoas utilizarem o sistema frequentemente, será muito mais fácil alcançar o suporte. Vários de nossos achados merecem uma discussão mais aprofundada. Nossos resultados sugerem que a aceitação da cobertura tele-UTI por parte da equipe geralmente é maior, apesar de os funcionários terem preocupações adequadas quanto à implementação e ao uso rotineiro dessa aplicação. É muito importante envolver a equipe antes da implementação e mitigar conflitos, como as preocupações quanto à responsabilidade, a carga de trabalho adicional e a impossibilidade de reconhecer quando essa ferramenta é necessária. Uma característica consistente dos programas de telemedicina que não foram bem-sucedidos é que eles se viam de forma separada da organização em geral e que tinham objetivos independentes. Esses programas perderam apoio ao longo do tempo ou foram relegados a um papel menor e muitas vezes experimental na prestação do cuidado.^(15,16)^

Antes da implementação do recurso, muitas práticas de cuidado eram ligadas à tomada de decisão médica, mesmo após tentativas de melhorar os processos, seja pela subutilização ou pela não adoção de protocolos ou pela transferência como um parâmetro. Portanto, ao comparar os resultados entre os pacientes que utilizaram esse recurso ou não, há uma tendência clara em favor de um melhor prognóstico e uma redução das transferências. Isso se deve à melhoria dos processos e a um maior envolvimento e comprometimento da equipe multidisciplinar.

A ferramenta da telemedicina não foi aplicada diretamente ao paciente. Este estudo envolveu fundamentalmente relação médico-médico, baseado em problema clínico envolvendo paciente. Este estudo não incluiu um questionário relacionado à satisfação do paciente. Realizaremos estudos futuros com essa finalidade.

A telemedicina é uma ferramenta, não um objetivo, e deve solucionar um problema real. A medicina ainda se trata de pessoas, pacientes, qualidade de serviços e processos. Garantir a continuidade do cuidado em um ambiente de telemedicina apresenta desafios únicos. São necessárias pesquisas para fornecer evidências das eficiências de custo e da melhoria da qualidade do cuidado por meio do uso da telemedicina para estados específicos de doença. Este estudo apresentou resultados iniciais encorajadores, e o programa provou ser útil no auxílio do diagnóstico, conduzindo casos críticos e transferindo conhecimento médico especializado para hospitais com falta de recursos humanos e técnicos.

O potencial total da telemedicina só será alcançado com mudanças na cultura médica e nas atitudes. Em um sistema dessa natureza, o compartilhamento de conhecimentos ocorreu e formou um novo paradigma na prestação de serviços de saúde por meio do qual organizações de saúde podem prestar serviços de alta qualidade a partir de áreas desenvolvidas para áreas menos desenvolvidas.

### Limitação

Nosso estudo teve algumas limitações importantes. Fizemos um projeto com a visão da telemedicina como uma plataforma para sistemas de telemedicina mais completos, tratando várias doenças além do acidente vascular cerebral em áreas em que o acesso ao cuidado especializado representava uma barreira importante à prestação de cuidados. Este é um estudo descritivo que analisa o impacto inicial da implementação do programa de telemedicina na qualidade e na segurança da prática clínica em um hospital comunitário em São Paulo (SP). A interpretação adequada dos dados de desfechos de pacientes exige informações de qualidade sobre as características dos pacientes, especialmente quanto ao seu estado de saúde. Comparações de intervenções ou programas clínicos devem ser ajustadas estatisticamente para levar em conta diferenças nos fatores de risco dos pacientes. Apesar de ter sido observada uma tendência de redução da taxa de mortalidade hospitalar no grupo de pacientes submetidos à consulta por telemedicina, os pacientes não foram comparados com relação aos índices de gravidade. Pode ser difícil conduzir um estudo prospectivo randomizado que compare dois grupos de pacientes, no que tange a problemas éticos e logísticos. Uma alternativa poderia incluir a comparação entre dois hospitais semelhantes, um deles com a telemedicina, estratificando o mesmo grupo de pacientes e de doenças.

## CONCLUSÃO

Este artigo apresentou um marco conceitual, e coletou alguns dados para provar e mostrar como a implementação da telemedicina pode ter papeis significativos na qualidade e na quantidade dos serviços, especialmente com relação ao conhecimento que pode ser transferido de todos os níveis da rede de saúde. A aplicação está associada a diferenças no uso dos serviços de saúde: transferências de emergência, mortalidade, implementação de protocolos e decisões quanto ao manejo dos pacientes, especialmente com relação à trombólise. Esses resultados destacam o papel da telemedicina como um vetor para transformação da cultura hospitalar, com impacto sobre a segurança e a qualidade do cuidado.
